# Human umbilical cord-derived mesenchymal stem cells alleviate schizophrenia-relevant behaviors in amphetamine-sensitized mice by inhibiting neuroinflammation

**DOI:** 10.1038/s41398-020-0802-1

**Published:** 2020-04-27

**Authors:** Min-Jung You, Minji Bang, Hyun-Sun Park, Bohyun Yang, Kyu Beom Jang, Jongman Yoo, Dong-Youn Hwang, MinYoung Kim, Borah Kim, Sang-Hyuk Lee, Min-Soo Kwon

**Affiliations:** 1 Department of Pharmacology, Research Institute for Basic Medical Science, School of Medicine, CHA University, CHA BIO COMPLEX, 335 Pangyo, Bundang-gu, Seongnam-si, Gyeonggi-do 13488 Republic of Korea; 2 Department of Psychiatry, CHA Bundang Medical Center, CHA University, Seongnam-si, Gyeonggi-do 13496 Republic of Korea; 3 Department of Microbiology, School of Medicine, CHA University, CHA BIO COMPLEX, 335 Pangyo, Bundang-gu, Seongnam-si, Gyeonggi-do 13488 Republic of Korea; 4 Department of Rehabilitation Medicine, CHA Bundang Medical Center, CHA University, Seongnam-si, Gyeonggi-do, 13497 Republic of Korea

**Keywords:** Stem cells, Stem cells, Schizophrenia, Schizophrenia

## Abstract

At present, therapeutic options available for treating schizophrenia are limited to monoamine-based antipsychotic drugs. Recent genome wide association study (GWAS) indicated a close relationship between immune system and schizophrenia. To leverage the GWAS finding for therapeutic strategy, we conducted a mechanism and effect study on application of human umbilical cord-derived mesenchymal stem cells (hUC-MSC) with potent immune-modulatory effect in an animal model useful for the study of schizophrenia. Schizophrenia-relevant behaviors were induced by amphetamine administration (amphetamine-sensitized mice) and the effect of a single intravenous administration of hUC-MSC was examined in the amphetamine-sensitized mice. Schizophrenia-relevant behaviors were assessed by open field test, light/dark box, social interaction test, latent inhibition, prepulse inhibition, tail suspension test, and forced swimming test. Our results indicated that neuroinflammation along with peripheral TNF-α elevation is associated with schizophrenia-relevant behaviors in amphetamine-sensitized mice. In addition, hUC-MSC inhibited schizophrenia-relevant and the neuroinflammatory changes. The main mechanism of hUC-MSC was associated with the induction of T_reg_ and production of the anti-inflammatory cytokine, IL-10 in periphery. In vitro study revealed that amphetamine did not directly induce a neuroinflammatory reaction, while recombinant TNF-α (rTNF-α) increased mRNA expression of TNF-α, KMO, and IL-1β in several microglial cell lines. Moreover, recombinant IL-10 (rIL-10) and MSC conditioned media inhibited the inflammatory response in rTNF-α-treated microglial cells. Assuming that hUC-MSCs rarely reach the CNS and do not remain in the body for an extended time, these findings suggest that a single hUC-MSC infusion have long-term beneficial effect via regulatory T cell induction and secretion of IL-10 in amphetamine-sensitized mice.

## Introduction

Schizophrenia is a serious, debilitating mental disorder associated with a substantial global health burden^1^. Following postulation of the dopamine hypothesis, treatment of schizophrenia has largely depended on anti-dopaminergic agents, which reduce mesolimbic dopamine transmission by blocking postsynaptic D2 receptors^2^. Although the currently available antipsychotic drugs (APDs) exert modest positive effects on symptomatic remission^3^, a definitive cure for schizophrenia is yet to be reported. Furthermore, APDs can cause considerable side effects, wherein the use of typical APDs may cause extrapyramidal symptoms, including rigidity, tremor, and tardive dyskinesia^4^, while the use of atypical ones have raised concerns regarding weight gain and metabolic dysregulation^5,6^. Therefore, the need to identify novel therapeutic targets for the treatment of schizophrenia is felt to be constantly increasing.

More recent studies have focused on the role of neuroinflammation in the pathogenesis and disease progression of schizophrenia. Epidemiological studies have suggested that maternal immune activation (MIA) caused by prenatal infections, may increase liability to schizophrenia by disrupting neurodevelopmental process^7,8^. The latest large-scale genome-wide association study (GWAS) showed a strong association between schizophrenia and major histocompatibility complex (MHC) genes, suggesting that immune dysregulation may contribute to an increased risk for schizophrenia^9^. A meta-analysis reported that pro-inflammatory cytokines, including interleukin (IL)-1β, IL-6, and tumor necrosis factor (TNF)-ɑ, were elevated in the peripheral blood of schizophrenia patients^10^. Postmortem analyses of the brain revealed that the density of microglia and innate immune cells in the central nervous system (CNS)^11^ was increased in chronic schizophrenia patients^12,13^. In vivo neuroimaging studies using positron emission tomography also provided supportive evidence for the presence of activated microglia in the gray matter of schizophrenia patients^14,15^ and individuals at ultra-high risk for psychosis^16^. Considering that neuroinflammation and immune dysregulation play crucial roles in the early pathogenesis of schizophrenia, which precedes the onset of full-blown psychosis^17^, it is felt that immune modulatory strategies may be useful for enhancing the treatment and course alteration of schizophrenia.

The use of minocycline, a potent inhibitor of activated microglia and induced neurotoxicity^18^, is reportedly beneficial, safe for use and helps to reduce clinical symptoms of schizophrenia patients as observed in recent meta-analyses^19,20^. Considering that pro-inflammatory cytokines released by activated microglia affect the function of the dopaminergic and glutamatergic systems adversely^21,22^, restoration of activated microglia may offer a means for treating schizophrenia. As a potent therapeutic alternative for the treatment of neuroinflammation, mesenchymal stem cells (MSCs), in conjunction with their differentiation and regenerative potential, have been suggested to possess immunomodulatory properties implicated in the treatment of several neurological and psychiatric disorders, including Alzheimer’s disease, multiple sclerosis, and autism^23–25^. MSCs are reportedly known to inhibit immune responses by promoting the production of anti-inflammatory cytokines (e.g., IL-10) and the generation of regulatory T-cell (*T*_reg_)^26–28^. Furthermore, MSCs regulate functional phenotypes of microglia, from an activated to an anti-inflammatory state, via secretion of the transforming growth factor (TGF)-β^29,30^. A placebo-controlled, crossover study using autologous umbilical cord blood stem cells of children with autism propose indirectly the therapeutic potential of MSCs targeting activated microglia and neuroinflammation^31^. However, to the best of our knowledge, experimental study is yet to support the clinical application of MSCs in schizophrenia patients.

The objective of the current study was to assess the effect of MSCs for reducing clinical symptoms of schizophrenia using mouse amphetamine sensitization models. The amphetamine-sensitized state is an animal model useful for the study of schizophrenia and has widely used to induce behavioral alterations indicative of psychotic symptoms, including hallucinations, delusions, and social defects^32^. We examined schizophrenia-related behaviors and the presence of activated microglia in amphetamine-sensitized mice, and analyzed the therapeutic effects of human umbilical cord blood-derived MSCs (hUC-MSC) in restoring behavioral aberrations and functional phenotypes of microglia. Finally, immunomodulatory mechanisms underlying the effects of hUC-MSC on microglia were demonstrated by in vitro experiments.

## Materials and methods

### Experimental animals

Male C57BL/6 mice aged 7–8 weeks (Orient Bio Inc. Seoul, Korea) weighing 25–30 g, were used for all experiments. Animals were housed in cages, each holding five animals, under SPF conditions at 22 ± 0.5 °C and an alternating 12-h light–dark cycle at the CHA BIO COMPLEX animal facility, and supplied with food and water ad libitum. Animals were allowed to acclimatize to the laboratory for 1 week before being subjected to experiments. To reduce variation, all experiments were performed during the light phase of the cycle. This study was approved by the Institutional Review Board at the CHA Bundang Medical Center for the use of umbilical cord (IRB, 2017-07-021). All experimental animals were manipulated in accordance with guidelines provided by the Institutional Animal Care and Use Committee of CHA University (IACUC180174).

### Amphetamine treatment and human UC-MSC infusion timeline

Mice were randomly assigned to control or experimental groups. To induce schizophrenia-relevant behaviors, amphetamine (Arlesheim, Switzerland) was injected (3XAMP injection) intraperitoneally three times (09:00, 13:00, and 18:00) at a dose of 1 mg/kg/day for six consecutive days, or administered three injections (09:00, 13:00, and 18:00) daily, using an escalating dose regimen consisting of 60 injections ranging from 1 to 10 mg/kg. Concurrently, only vehicle was injected into control group mice. Next, hUC-MSC was infused intravenously 1 day following the final amphetamine injection, and then, a series of behavior assessments were performed.

### Behavioral testing

Mice were allowed to acclimate to a testing room for at least 30 min before performing the assessments. All assessments were conducted during the light cycle between 9:00 AM and 4:00 PM. The open field test (OFT), light/dark test (LD), tail suspension test (TST), forced swim test (FST), three chamber social interactions (SI), latent inhibition (LI), and prepulse inhibition (PPI) were performed in a series. The order of these behavioral assays was changed slightly, depending on experimental design. Each assessment was conducted in 1 day as per our previous studies^33,34^. Each behavior test was described in supplementary information. Observers were blinded to the groups and measured data were compared by two observers to minimize bias.

### Preparation of human UC-MSC

All information pertaining to subjects and all human samples were used in compliance with Korean legislation, and all human participants provided informed written consent. The hUC-MSC (Cat# C-M03-W01 and Cat# C2-M02-W01-P003) was purchased from CHA Biotech, Co. Ltd. (Seongnam, Korea). All manufacturing and product testing procedures for the generation of Cordstem were performed under good manufacturing practice (GMP) conditions^35^. The preparation and characterization of cells have been described previously^35^. The cells were incubated under hypoxic conditions (3% O_2_, 5% CO_2_, and 37 °C) and stocked at a concentration of 2 × 10^7^cells/ml at the fourth passage. Stocked hUC-MSC was utilized for our experiment. The stocked hUC-MSC was washed with PBS and diluted with normal saline. After checking cell viability, the hUC-MSC (1.25 × 10^5^ cells in 100 µL of normal saline) were slowly administered to mice and the number was tolerable to mice in our study. The number hUC-MSC was determined to be highest number that did not cause death.

### Immunohistochemistry

For perfusion purposes, mice were sacrificed following behavioral assessment. The mice were deeply anesthetized using pentobarbital (100 mg/kg, i.p.), and perfused intracardially with physiological saline followed by ice-cold phosphate-buffered 4% paraformaldehyde (pH 7.4). Each brain was dissected and post-fixed in the same fixative for 4 h at 4 °C. Next, the brain blocks were cryoprotected in 30% sucrose for 24 h at 4 °C. Twenty-five millimeter thick sections were obtained using an electronic cryotome. Immuno-histochemical staining was performed with an Elite ABC Kit (Vector Laboratories). Sections were first rinsed thrice with 0.1 M bovine serum albumin, for 10 min each time, and pre-incubated in 0.1 M PBS containing 1% bovine serum albumin and 0.2% Triton X-100 for 30 min. After rinsing twice with 0.1 M PBS containing 0.5% BSA for 10–15 min each time, sections were incubated with polyclonal anti-rabbit anti-Iba-1 antibodies (1:300; Wako, # 019-19741) diluted with 0.1 M PBS containing 0.5% BSA and 0.05% sodium azide at room temperature. Following an overnight incubation, sections were rinsed and incubated with biotinylated anti-rabbit IgG secondary antibodies (Vector), 1:200 diluted with 0.1 M PBS containing 0.5% BSA for 1 h at 20–25 °C. After rinsing, the sections were incubated with ABC reagent, diluted 1:200 with PBS for 1 h at 20–25 °C, and rinsed with PBS followed by 0.1 M phosphate buffer. Finally, sections were incubated in a SIGMA FAST DAB kit (Sigma) until the desired stain intensity developed. Sections were rinsed with 0.05 mol/L phosphate buffer, dehydrated using an ascending ethanol gradient, cleared in histoclear (Fisher) and cover slipped using Permount (Fisher).

Histological analysis was modified from a previous study^33^ as follows: the number of cells that were immunoreactive to Iba-1 was counted by two blinded observers using a microscope (Nikon); the number of immunoreactive (IR) cells in the prefrontal cortex (interaural 6.14 mm, bregma 2.34 mm), striatum (interaural 4.78 mm, bregma 0.98 mm) and hippocampus (interaural 2.10 mm, bregma 1.70 mm) were counted in three sections from each mouse with reference to the mouse atlas. Counting was repeated in order to reduce counting bias. The number of animals in each group was five, and the entire process was repeated thrice independently.

### Quantitative reverse transcriptase polymerase chain reaction (qRT-PCR)

The striatum, hippocampus, and mesenteric lymph node were dissected, following behavioral assessments, to analyze mRNA expression. For RNA extraction, frozen tissue was homogenized in 1 mL of QIAzol reagent per 100 mg of tissue (Qiagen, Valencia, CA). Chloroform was added to separate the phase containing RNA, and isopropyl alcohol was added to precipitate RNA. Each precipitated RNA pellet was air-dried and redissolved in DEPC-treated water (Bioneer, Seongnam, Korea). Quantification of RNA concentration was determined by measuring absorption at 260 nm. One µg of messenger RNA (mRNA) was reverse-transcribed into cDNA in 20 μL of reaction mix using a RevertAid First Strand cDNA Synthesis kit (Thermo Scientific). Quantitative PCR was performed using Power SYBR® Green PCR Master Mix (Life technologies, Warrington, UK). Primer sequences are listed (Table S1). Cyclic conditions consisted of initial enzyme activation at 95 °C for 5 min, followed by 40 cycles of denaturation at 95 °C for 20 s, annealing, and extension including detection of SYBR Green bound to PCR product at 56 °C for 40 s. Glyceraldehyde 3-phosphate dehydrogenase (GAPDH) was used as an internal control for normalization. The relative quantities of PCR fragments were calculated using the comparative CT method.

### Total protein extraction and western blot analysis

Striatum protein was extracted and expression levels were assessed using western blotting. After dissecting the striatum, the tissue was washed twice with cold Tris-buffered saline (TBS; 20-mM Trizma base and 137 mM NaCl, pH 7.5). Immediately after washing, cells were lysed with SDS lysis buffer (62.5-mM Trizma base, 2% w/v SDS, 10% glycerol) containing 0.1 mM Na_3_VO_4_, 3 mg/mL aprotinin, and 20 mM NaF. After a brief sonication to shear DNA and reduce viscosity, protein concentration was determined with a detergent-compatible protein assay reagent (Bio-Rad Laboratories), using bovine serum albumin as the standard. After adding dithiothreitol (5 mM) and bromophenol blue (0.1% w/v), the proteins were boiled, separated by electrophoresis in 10–16% polyacrylamide gels (Invitrogen) and transferred onto a polyvinylidene difluoride (PVDF) membranes (Bio-Rad Laboratories). The membranes were blocked on a shaker for 1 h at room temperature. Blocking buffer consisted of TBST (Tris-buffered saline/0.1% Tween-20) and 5% skim milk. Primary antibodies were dissolved in the blocking buffer and the membranes were immunoblotted with anti- bodies against Dopamine 2 receptor (D2R 1:200, Bioss #bs-1008R), dopamine transporter (DAT, 1:200, abcam#ab111468), tyrosine hydroxylase (1:200, abcam#ab112), glutamate decarboxylase 67 (GAD67, 1:500, #ab26116), PSD95 (1:300, #ab18258), synaptophysin (1:500, #ab14692) and beta-actin (1:1000, Cell Signaling). The membranes were incubated in goat anti-rabbit (1:1000, Enzo #ABI-SAB-300-J) or goat anti-mouse (1:1000, BETHYL, A120-101P) and dissolved in blocking buffer at a room temperature for 1 h. The membranes were visualized with ECL-plus solution (Amersham Pharmacia Biotech). The membranes were then exposed to chemiluminescence (LAS- 4000, Fujifilm) for detection of light emission. Western blot results were quantified using ImageJ 1.51 software (National Institutes of Health, Bethesda, MD) following the densitometric scanning of films.

### Cytokine assay

Mouse serum and supernatant media cytokine levels such as TNF-α, IL-6, IL-4, IL-10, and IFN-γ were measured using Bio-Rad Bio-Plex® assay (Bio-Rad, Hercules, CA) according to the manufacturer’s instruction.

### SIM-A9 culture

SIM-A9 is a microglial cell line purchased from Kerafast (Boston, USA). These cells, referred to as SIM-A9 cells and relate to native primary microglial cells, have been characterized for both morphology and release of cytokines/chemokines. Upon receiving, the cells were passaged in an uncoated 100 mm cell culture dish in DMEM/F-12 (Gibco, cat. # 11320-033) containing 10% heat-inactivated fetal bovine serum (gibco, cat. # 16000-044), 5% heat-inactivated horse serum (Invitrogen cat. # 16050-122), and 1% penicillin/streptomycin (Gibco, cat. # 15140122). Cells were cultured at 37 °C in a humidified incubator with 5% CO_2_. Following serum starvation overnight, the cells were treated with microglial media (control) or 10 μg/mL of amphetamine for 12 h. To investigate the effect of TNF-α, the cells were starved overnight and the whole culture medium was mixed with recombinant TNF-α (rTNF-α, Prospec, cat. # Cyt-252) at various concentrations (0, 0.01, 0.05, 0.1, 0.5, 1 μg/mL) for 6, 12, 18, or 24 h. TNF-α treatment group in SIM-A9 was defined as 0.1 μg/mL of treatment for 12 h. Next, to investigate the effect of hUC-MSC conditioned media (MSC-CM) on the TNF-α treated group, rTNF-α treatment was treated for 6 h in SIM-A9, following which the media was changed and new media, MSC media or MSC-CM was treated with rTNF-α alone, rTNF-α+MSC media, rTNF-α+MSC-CM or rTNF-α+MSC-CM+IL-10Ab for 6 h.

To investigate the effect of recombinant IL-10 (rIL-10) on the TNF-α treated group, rTNF-α was treated for 6 h in SIM-A9 and the media was changed and new media alone, media with 10 ng/mL of rIL-10 (R&D, # 417-ML), 10 ng/mL of rIL-10 with 100 ng/mL of IL-10Ab or 500 ng/mL of IL-10Ab (R&D, # MAB417-SP) was added with rTNF-α for an additional 6 h. Other methods for other microglial cell line culture were described in supplementary information.

### Human UC-MSC-conditioned media

Remaining cells after in vivo experiment and another hUC-MSC line (Cat# C2-M02-W01-P003) were used for MSC-CM manufacture. MSC-CM was collected according to the procedure described in a previous study of ours^30^. Briefly, cells were cultured at a concentration of 1 × 10^4^ cells per cm^2^ in MEM α with Glutamax™ (gibco, cat. # 32561037) and supplemented with 10% FBS and 5 mg/mL gentamicin (ThermoFisher Scientific) at 37 °C with 5% CO_2_. The medium was refreshed twice a week, and the cells were subcultured before reaching 70–80% confluence. hUC-MSC (5 × 10^5^ cells/mL, passage 7) were plated into 6-well plates (9.5 cm^2^) in serum-free culture media. After 24 h, the medium was replaced, and the cells were incubated for another 48 h, following which the medium was collected and centrifuged at 1500 rpm for 10 min at 4 °C and filtered through 0.22 mm filters (Merck Millipore, Billerica, MA) prior to being used. MSC-CM obtained from two hUC-MSC lines were collected together. The medium was defined and used as MSC-CM in our study.

### Statistical analysis

The outliers were excluded and the data within mean ± 2 SD were included for calculation. Data are presented as mean ± standard error of the mean (SEM). The statistical significance of differences between groups was assessed with Student’s *t*-test and one-way or two-way analysis of variance (ANOVA) using GraphPad Prism version 7 for Mac (GraphPad, La Jolla, CA). Tukey’s post hoc test was performed in one-way ANOVA when *p* values were <0.05. *p* < 0.05 was considered as statistically significant.

## Results

### Amphetamine-sensitized mice by 3XAMP showed social deficit and disruption of latent inhibition

**Fig. 1:**
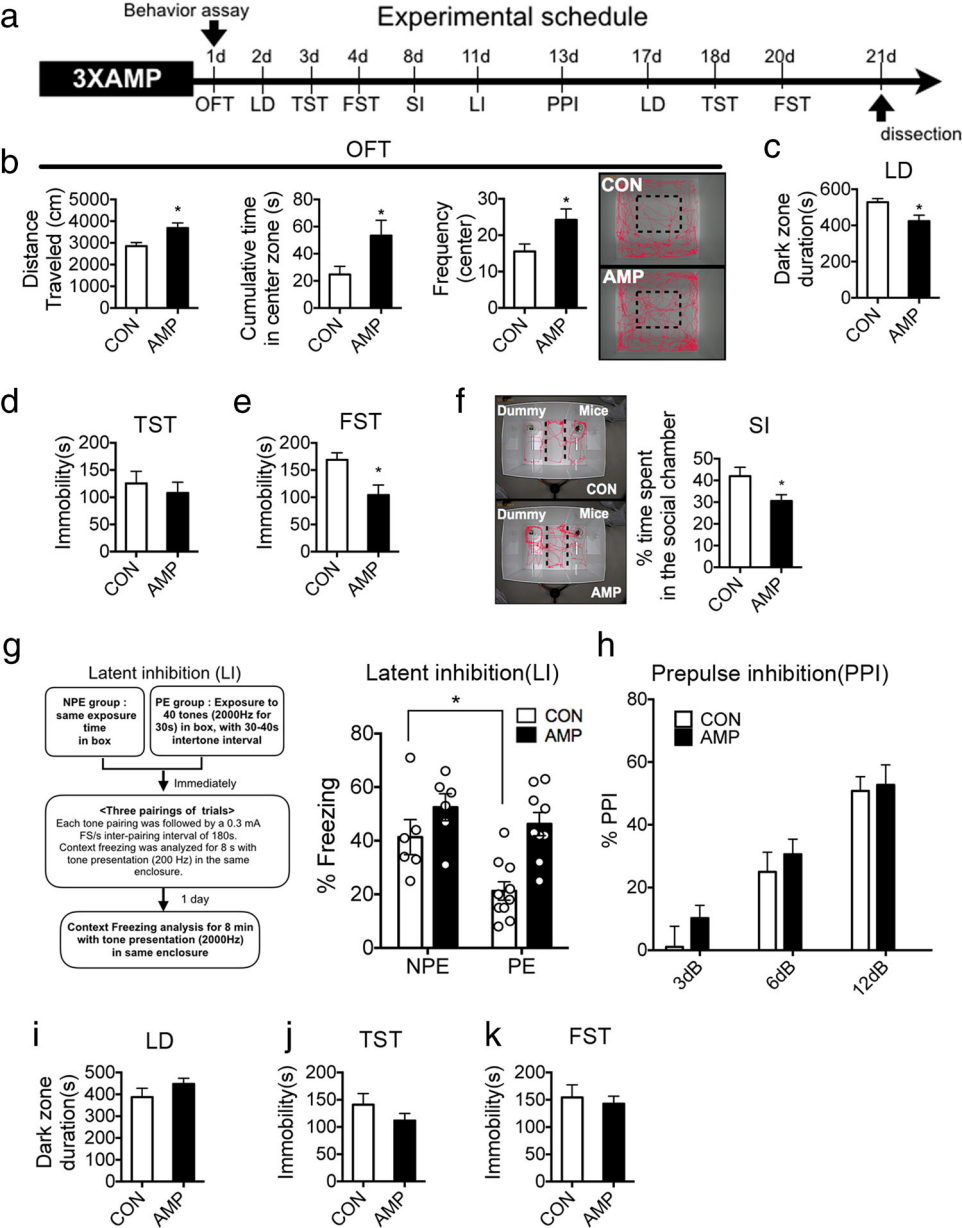
Behavioral profiles in amphetamine-sensitized mice induced by 3XAMP regimen. **a** 1 mg/kg of amphetamine was administered intraperitoneally three times per day for 6 days (3XAMP) for induction of schizophrenia-relevant behaviors and a series of behavioral assessments for 21 days was performed in the amphetamine-sensitized mice (AMP). Vehicle was administered to control group (CON) as same regimen. **b** AMP showed mania-like behavior (longer distance traveled, much cumulative time in center zone, and more frequency in center zone) in an open field test (OFT), **c** stayed longer in the light region in the light–dark box test (LD), reduced immobility time in **d** tail suspension test (TST), and **e** forced swimming test (FST) compared to CON. **f** AMP spent much time in dummy chamber in the three chambers social interaction test (SI). **g** AMP showed more freezing behavior in pre-exposed group (PE), but there was no difference between CON and AMP in the non pre-exposed group (NPE). **h** AMP induced by the 3XAMP regimen did not show any difference compared to CON in the pre-pulse inhibition test (PPI). Mania-like behaviors initially seen after the withdrawal of amphetamines was normalized in LD (**i**), TSF (**j**), and FST (**k**). *n* = 8–10 in each group *p < 0.05, **p < 0.01 compared with the CON. LI was performed 2 times independently; *n* = 14–15 per CON or AMP. The data shown are mean ± standard error of the mean (SEM). *p < 0.05 compared to CON of NPE, AMP effect was significant (*F*_(1,27)_ = 7.738, *p* = 0.0097) on latent inhibition.

In order to induce schizophrenia-relevant behaviors, mice were treated with amphetamine as a 3XAMP regimen, and behavioral patterns following amphetamine withdrawal were determined through a series of behavioral assessments (Fig. 1a). In behavioral experiments performed within a week after amphetamine withdrawal, mice displayed mania-like behavior. In the OFT, amphetamine-sensitized mice showed an increase in locomotor activity, residence time as well as in the number of entries to the central zone (Fig. 1b). In addition, the time spent in the dark zone by amphetamine-sensitized mice in the LD test was lower than that in the control group (Fig. 1c), and immobility time was also decreased in TST (Fig. 1d) and FST (Fig. 1e). It was found that sociability was reduced, and latent inhibition impaired in the SI (Fig. 1f) and LI (Fig. 1g) behavioral experiments, which were conducted 2–3 weeks later. However, no difference was observed in the PPI test (Fig. 1h), and the mania-like behavior observed in the first week disappeared in the resumed LD (Fig. 1i), TST (Fig. 1j) and FST (Fig. 1k) at 3 weeks.

### Amphetamine increased Iba-1 positive cells and changed microglia functional phenotypes in the striatum and the hippocampus

**Fig. 2:**
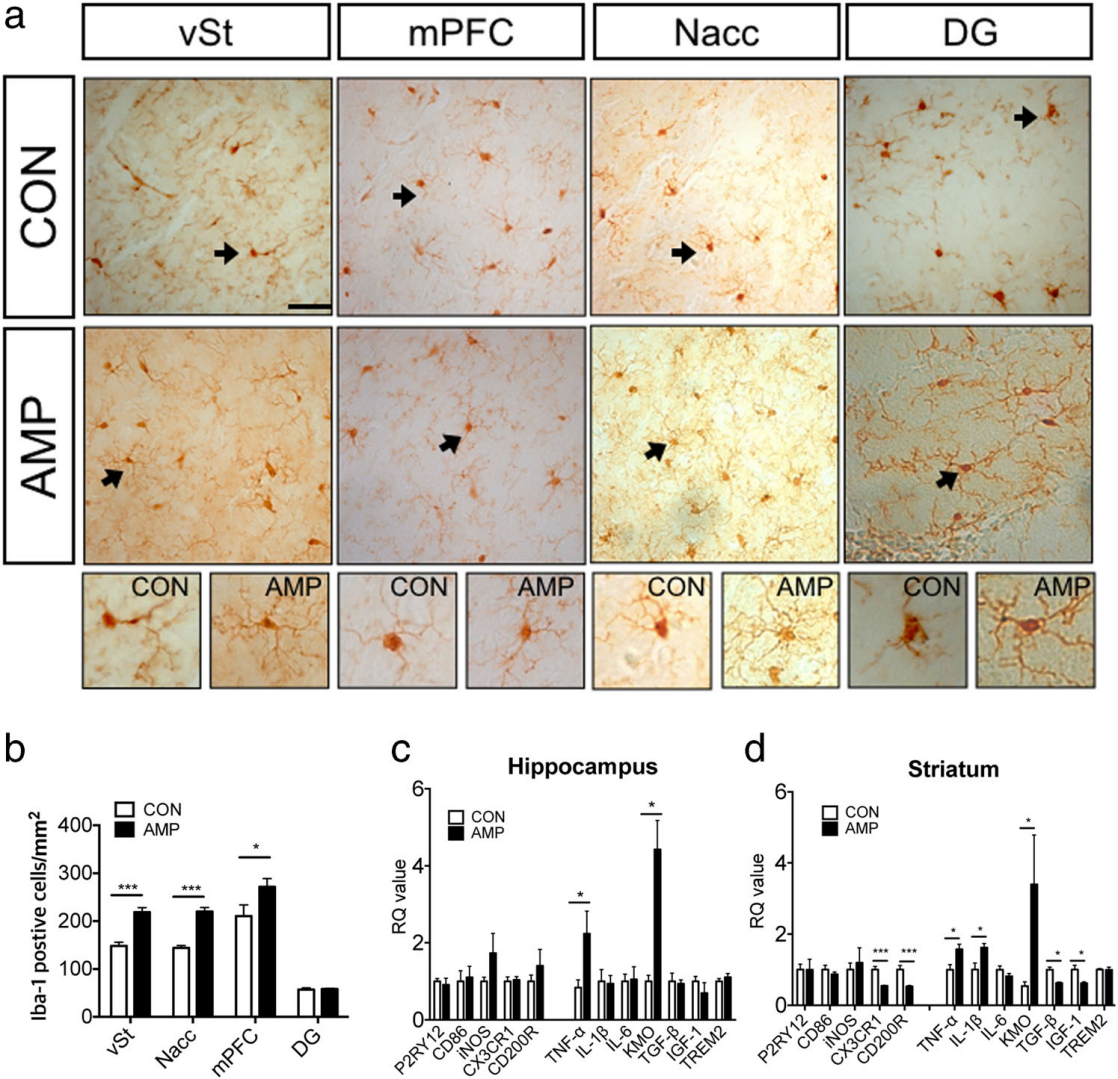
Neuroinflammation in schizophrenia-relevant regions of amphetamine-sensitized mice. **a** An immunohisotochemical study was performed in order to determine morphology and number of Iba-1 positive cells in medial prefrontal cortex (mPFC), ventral region of striatum (vSt), Nucleus accumbens (Nacc) and dentate gyrus (DG) of hippocampus. **b** To quantify, we counted Iba-1 positive cells in vST, Nacc, mPFC, and DG. Microglia-related factors, which are functional phenotype markers (P2RY12, CD86, iNOS, CX3CR1, CD200R, and TREM2) and cytokines (TNF-α, IL-1β, KMO, TGF-β, and IFG-1), were examined in the hippocampus (**c**) and the striatum (**d**) using qPCR. *n* = 3 in each group in immunohistochemistry. For qPCR, *n* = 8–10 per group and the data shown as mean ± standard error of the mean (SEM), *p < 0.05, ***p < 0.001 compared with CON. Scale bar is 10 μm.

We performed immunohistochemical staining of microglial cells at brain sites associated with schizophrenia-relevant behaviors in amphetamine-sensitized mice treated with the 3XAMP regimen following a series of behavioral assessments. Microglial cells displayed a hyper-ramified form with prolonged processes and increased bifurcation of processes in the ventral region of the striatum, the medial prefrontal cortex, the nucleus accumbens, and the dentate gyrus (Fig. 2a). It was also confirmed that the number of microglial cells was increased in all the observed regions except dentate gyrus (Fig. 2b). Changes in factors related to microglial cell activity were confirmed by qPCR, where it was observed that TNF-α and KMO mRNA levels were increased in the hippocampus (Fig. 2c). In the striatum (Fig. 2d), the anti-inflammatory phenotypes, CX3CR1, and CD200R, decreased, as did the anti-inflammatory cytokines, TGF-β and IGF-1. Pro-inflammatory markers, such as TNF-α, KMO, IL-1β, were also increased in the striatum.

### Intravenous human UC-MSC infusion alleviated schizophrenia-relevant behaviors and reduced the increase in Iba-1 positive cells in amphetamine-sensitized mice

**Fig. 3:**
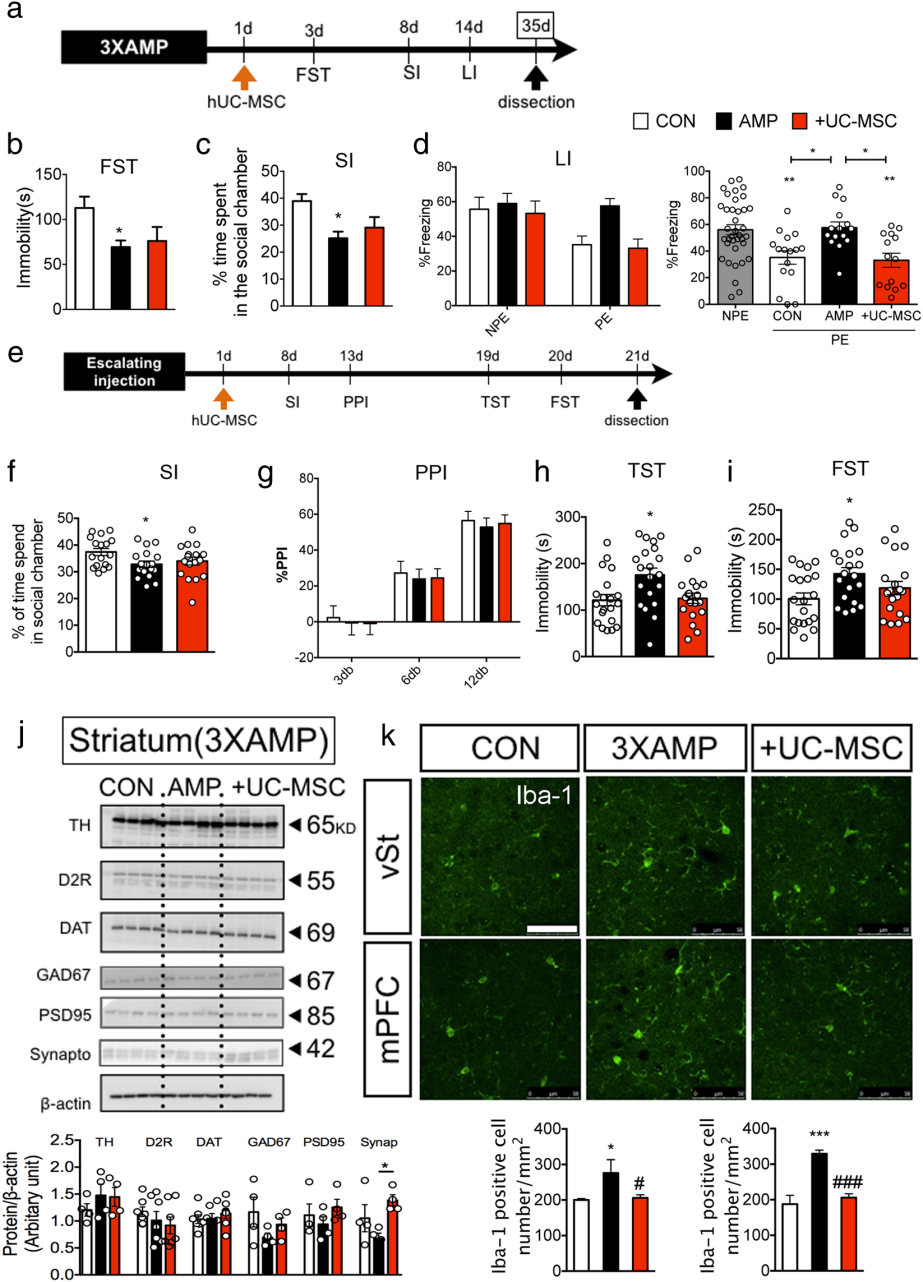
Behavioral rescue of intravenous human UC-MSC infusion in amphetamine-sensitized mice. **a** Intravenous human UC-MSC was infused intravenously 1 day following the final 3XAMP injection after which FST, SI, and LI were performed. Mice were sacrificed at 35 days following hUC-MSC treatment. In behavioral assessments, hUC-MSC did not restore reduced immobility time in FST (**b**) and social deficit in SI (**c**), but rescued disruption of LI (**d**) in 3XAMP treated mice. *n* = 10 in FST and SI, LI test was performed three times independently, *n* = 27–30 per group. In another amphetamine treatment regimen (escalating regimen) (**e**), Sociality was impaired (**f**) and PPI was not changed in all groups (**g**) hUC-MSC reduced immobility time in TST (**h**) and FST (**i**) in amphetamine-sensitized mice by escalating regimen. TST and FST was performed twice independently *n* = 19–20 per group. **j** Tyrosine hydroxylase (TH), dopamine 2 receptor (D2R), dopamine transporter (DAT), glutamate decarboxylase 67 (GAD67), PSD95, and synpatophysin were assessed by western blot analysis and their expression was quantified using Image J in 3XAMP regimen mice, with *n* = 4 per group. **k** An immunofluorescence study was performed to determine the effect of hUC-MSC on number of Iba-1 positive cells in the ventral region of the striatum (vSt) and the medial prefrontal cortex (mPFC) and was quantified using Image J in in the 3XAMP regimen mice; *n* = 3 per group. Data are shown as mean ± standard error of the mean (SEM); scale bar is 50 μm. *p < 0.05, **p < 0.01. ***p < 0.001 compared with the CON or NPE. ^#^*p* < 0.05 compared with the AMP.

After analyzing behavioral and molecular changes in amphetamine-sensitized mice and related brain regions, we hypothesized that hUC-MSC, which regulates microglia functional phenotypes and the peripheral immune system, may contribute to the alleviation of the abnormal behaviors. hUC-MSC was intravenously administered 1 day after the final administration of 3XAMP, following which a series of behavioral experiments was performed (Fig. 3a). hUC-MSC was not effective in restoring mania-like behavior in FST (Fig. 3b) and sociality (Fig. 3c) in amphetamine-sensitized mice. However, it was confirmed that disruption of latent inhibition was fully rescued by hUC-MSC (Fig. 3d). In another AMP regimen (escalating injection, Fig. 3e), which induced social deficit and depressive-like behavior, hUC-MSC did not have an effect on social deficit like 3XAMP regimen (Fig. 3f) and did not induce PPI impairment in a manner similar to that induced by the 3XAMP regimen, and thus the effect of hUC-MSC on PPI in amphetamine-sensitized mice could not be evaluated (Fig. 3g). However, hUC-MSC exhibited an antidepressant effect (Fig. 3h, i). Following behavioral assessments, we speculated whether the effects of hUC-MSC were associated with the dopamine system in the striatum. The 3XAMP regimen did not change tyrosine hydroxylase (TH), dopamine 2 receptor (D2R), dopamine transporter (DAT), GAD67, and PSD95 in western blot. Synpatophysin was normalized by hUC-MSC in amphetamine-sensitized mice (Fig. 3j). In this experimental schedule (3XAMP), mice were sacrificed at 35 days instead of 21 days in order to evaluate whether the changes in microglia-related factors were persistently maintained (Fig. 2). Microglia activation was persistent at 35 days in 3XAMP and hUC-MSC inhibited the change as demonstrated by the immunofluorescence study (Fig. 3k).

### Intravenous human UC-MSC infusion restored alteration of cytokines induced by amphetamine in the striatum and hippocampus

**Fig. 4:**
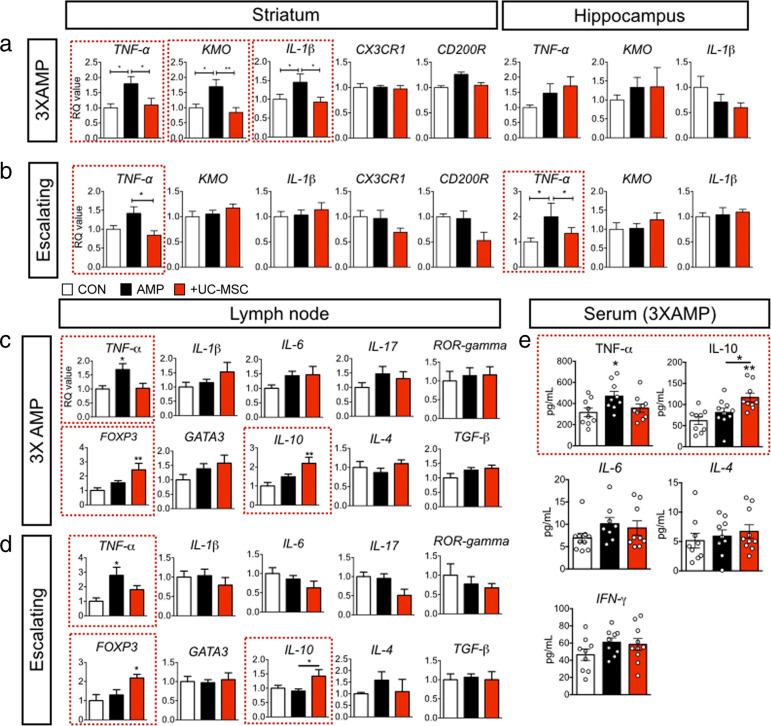
Anti-inflammatory effect of human UC-MSC along with immune-modulation in amphetamine-sensitized mice. The mRNA expression of activated microglia-related markers in the striatum and the hippocampus of amphetamine-sensitized mice by 3XAMP (**a**) or escalating regimen (**b**) were measured using qPCR. CT values were normalized as a ratio where controls were 1 and RQ value referred to the ratio of respective transcription factors as a percentage of the controls; *n* = 8 per group. Data are shown as mean ± standard error of the mean (SEM). *p < 0.05, **p < 0.01 compared with CON or +UC-MSC. The mRNA expressions of peripheral cytokines and T cell subtype markers in mesenteric lymph node of amphetamine-sensitized mice by 3XAMP (**c**) or escalating regimen (**d**) were measured using qPCR. CT values were normalized as a ratio where controls were 1 and RQ value referred to the ratio of respective transcription factors as a percentage of the controls; *n* = 8 per group. The data are shown as mean ± standard error of the mean (SEM). *p < 0.05, **p < 0.01 compared with CON or +UC-MSC. **e** Serum of each group was obtained to measure TNF-α, IL-10, IL-6, IL-4, and IFN-γ using Bio-Rad Bio-Plex® assay; *n* = 7–8 per group. Data are shown as mean ± SEM. *p < 0.05, ***p < 0.001 compared with the CON or AMP.

Next, we performed qPCR to determine whether the inflammatory response factors, which were changed in the striatum and hippocampus by amphetamines, were regulated by hUC-MSC. Both regimens, 3XAMP (Fig. 4a) and escalating regimen (Fig. 4b), commonly increased TNF-α in the striatum, and this increase was inhibited by hUC-MSC. The increase in KMO and IL-1β mRNA in the 3XAMP regimen were also inhibited by hUC-MSC. However, 3XAMP did not affect cytokine alteration in the hippocampus compared to the escalating AMP regimen. Compared to the 3XAMP regimen, escalating AMP regimen increased TNF-α in the hippocampus and this was also inhibited by hUC-MSC. Besides, we observed that the decreased CX3CR1, CD200R, TGF-β, and IGF-1 at 21 days following amphetamine withdrawal in the striatum were recovered at 35 day, in contrast to TNF-α, KMO, and IL-1β.

### Intravenous human UC-MSC commonly inhibited TNF-α and increased IL-10 with FOXP3 mRNA elevation in mesenteric lymph node and serum

Peripheral inflammatory cytokines may also affect microglia activation and schizophrenia-relevant behaviors. To evaluate alteration of the peripheral immune system, we examined cytokine changes in the lymph nodes in both 3XAMP (Fig. 4c) and escalating regimens (Fig. 4d) with particular reference to the relationship between the peripheral immune system and microglia activation. TNF-α mRNA levels were commonly increased in both regimens, where these increases were inhibited by hUC-MSC. In addition, the mRNA expression levels of IL-10, an anti-inflammatory cytokine, and FOXP3, a marker of regulatory T cells, were increased in the hUC-MSC treated group. Circulating TNF-α and IL-10 levels showed patterns that were similar to those of the qPCR result (Fig. 4e). However, other cytokines such as IL-6, IL-4, and IFN-γ was not changed. There was no change in the other pro-/anti-inflammatory cytokines and T cell subtype markers.

### In vitro study recapitulates the possible mechanism of hUC-MSC on amphetamine-sensitized mice

**Fig. 5:**
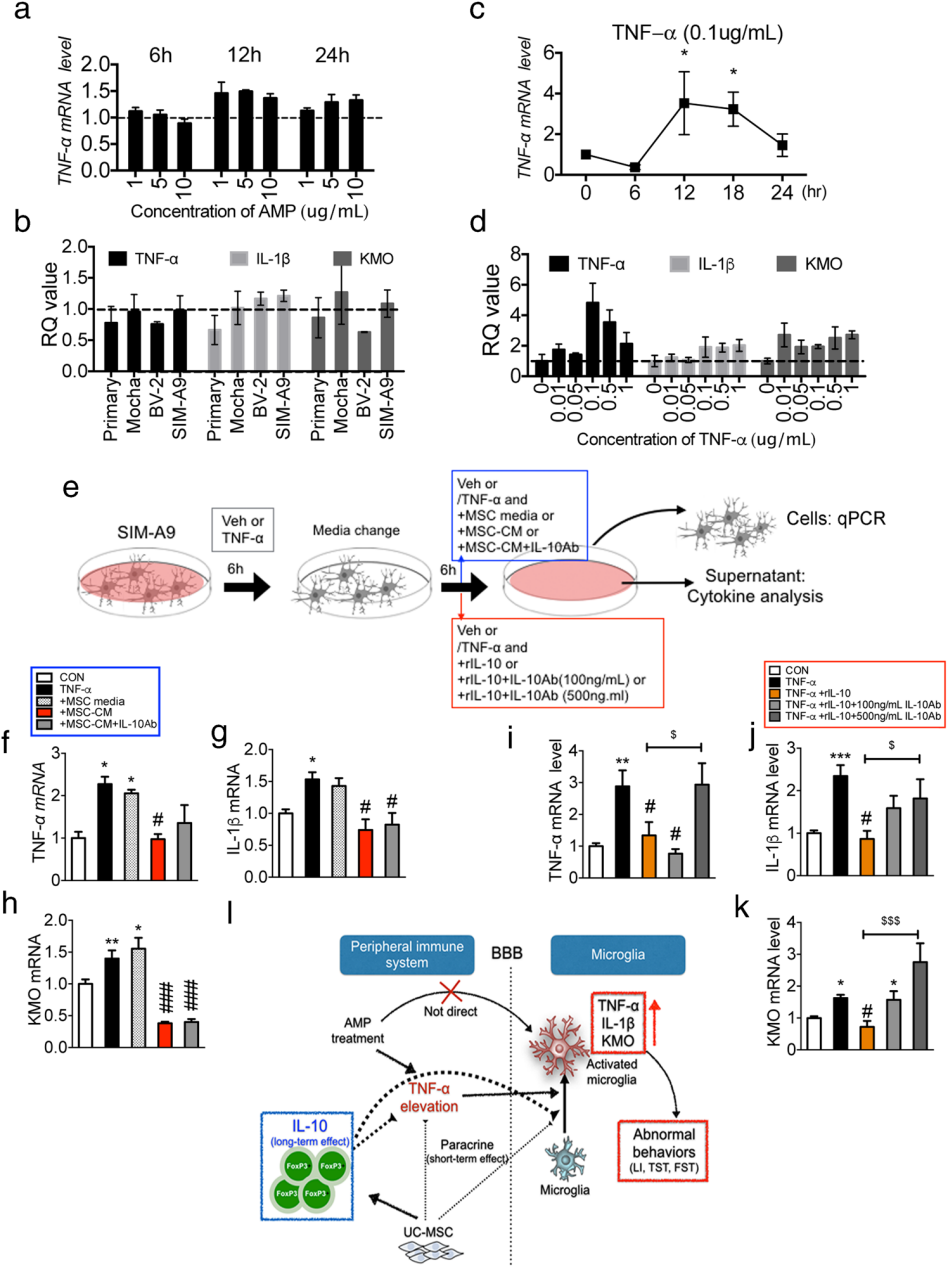
Possible mechanism of hUC-MSC through in vitro experiment. **a** Microglial cell lines (SIM-A9) were treated with various concentrations (0, 1, 5, 10 μg/mL) of amphetamine (AMP) for 6, 12, or 24 h and mRNA levels of TNF-α were measured by qPCR. **b** Rat primary cultured microglia, Mocha, BV-2 and SIM-A9 microglia cell lines were treated with 10 μg/mL AMP for 12 h and mRNA levels of TNF-α, IL-β and ΚΜΟ were measured using qPCR. **c** Time-course effect of recombinant TNF-α (rTNF-α) on TNF-α mRNA level was examined in the SIM-A9 cell line. **d** SIM-A9 was treated with various concentrations of 0, 0.01, 0.05, 0.1, 0.5, and 1 μg/mL of rTNF-α for 12 h and mRNA levels of TNF-α, IL-β and ΚΜΟ were measured using qPCR. **e** SIM-A9 was treated with vehicle (Veh) or rTNF-α (0.1 μg/mL) for 6 h and then media was changed to media mixed Veh or mixed with rTNF-α (0.1 μg/mL) or to MSC media mixed rTNF-α (0.1 μg/mL) or MSC-CM mixed rTNF-α (0.1 μg/mL), or MSC-CM mixed rTNF-α (0.1 μg/mL)+IL-10 neutralizing antibody (IL-10Ab) (Blue box design). In the red box design, after change of media, media mixed with Veh or with rTNF-α (0.1 μg/mL) or mixed with rTNF-α (0.1 μg/mL)+recombinant IL-10 (rIL-10) or rTNF-α (0.1 μg/mL)+rIL-10 + 100 ng/mL of IL-10Ab, or rTNF-α (0.1 μg/mL)+rIL-10 + 500 ng/mL of IL-10Ab was added. Next, the supernatant and cells were separated for cytokine analysis and qPCR, respectively. In the blue box design, MSC-CM inhibited the increased mRNA level of TNF-α (**f**), IL-1β (**g**), and KMO (**h**) in rTNF-α-treated SIM-A9. IL-10Ab did not abolish the effect of MSC-CM. In the red box design, rIL-10 inhibited the increased mRNA level of TNF-α (**i**), IL-1β (**j**), and KMO (**k**) in rTNF-α-treated SIM-A9. IL-10Ab abolished the effect of rIL-10. **l** Schematic illustration of the entire mechanism of hUC-MSC in amphetamine-sensitized mice based on our current results. The in vitro study was performed three times independently; *n* = 3. The data are shown as mean ± SEM. *p < 0.05, **p < 0.01, ***p < 0.001 compared with the CON. ^#^*p* < 0.05, ^###^*p* < 0.001 compared with the rTNF-α. ^$^*p* < 0.05, ^$$$^*p* < 0.001 compared between two groups.

Based on our in vivo results, we designed follow up in vitro experiments to address the cause of persistent neuroinflammation despite amphetamine withdrawal and to elucidate mechanisms underlying the effect of single intravenous hUC-MSC on the peripheral immune system, focusing on the elevation of regulatory T cells and IL-10. First, we examined the effect of amphetamine on increased TNF-α, IL-1β, and KMO mRNA levels in microglial cells. The microglial cell line, SIM-A9, was treated with 1, 5, and 10 μg/mL of amphetamine (AMP). The change in TNF-α mRNA was examined at 6, 12, and 24 h following AMP treatment and it was observed that AMP alone did not affect microglial cell activation (Fig. 5a). AMP (10 μg/mL for 12 h) did not affect IL-1β, KMO, and TNF-α mRNA levels in primary cultured microglia cell lines or other cell lines (Fig. 5b). Therefore, we concluded that AMP does not directly increase the inflammatory response in microglial cells. Next, we hypothesized that increased TNF-α in the periphery may be associated with neuroinflammation in the amphetamine-sensitized mice. Thus, SIM-A9 cell line was treated with 0.1 μg/mL recombinant TNF-α (rTNF-α) and TNF-α mRNA levels were determined following 6, 12, 18, and 24 h (Fig. 5c), and results indicated that rTNF-α significantly increased TNF-α mRNA levels at 12 h. Next, to investigate the dose-dependent effect of rTNF-α, SIM-A9 was treated for 12 h with 0.01, 0.05, 0.1, 0.5, and 1 μg/mL of rTNF-α following which TNF-α, IL-1β, and KMO mRNA levels were examined (Fig. 5d). We found that treating with rTNF-α (0.1 μg/mL) effectively increased TNF-α, IL-1β, and KMO mRNA levels in 12 h. After optimizing concentrations and time points for rTNF-α treatment in SIM-A9, we examined the effect of MSC-CM and recombinant IL-10 (rIL-10) on TNF-α-treated SIM-A9. The experimental design is described (Fig. 5e). MSC-CM inhibition of TNF-α (Fig. 5f), IL-1β (Fig. 5g), and KMO (Fig. 5h) mRNA levels in TNF-α-treated SIM-A9 was not mediated by IL-10. Additionally, IL-10 concentration in MSC-CM was rarely detectable (Table S2). Moreover, rIL-10 also exerted an effect very similar to that of MSC-CM and this effect was abolished by IL-10 which neutralized Ab (IL-10Ab); (Fig. 5i–k). Based on these results, we concluded that microglial activation and neuroinflammation in amphetamine-sensitized mice may involve elevation of circulating TNF-α rather than a direct effect of amphetamine, and that IL-10 may play a crucial role in inhibiting microglia activation and schizophrenia-relevant behaviors seen in amphetamine-sensitized mice (Fig. 5l).

### rIL-10 infusion was not enough to inhibit schizophrenia-relevant behaviors in amphetamine-sensitized mice

After confirming that rIL-10 mirror the possible mechanism of MSC in vitro, we wondered whether intravenous IL-10 infusion also inhibited schizophrenia-relevant behaviors in amphetamine-sensitized mice. To address this issue, recombinant IL-10 (rIL-10, 100 ng per day)^34^ was infused intravenously next day after amphetamine withdrawal for 10 days and then LI test was performed in 3XAMP. TST and FST also were conducted in escalating AMP regimen. However, as shown in Supplementary Fig. 1, rIL-10 infusion was not enough to inhibit schizophrenia-relevant behaviors in amphetamine-sensitized mice.

## Discussion

The current study demonstrated that amphetamine-sensitized mice showed sustained inflammation in their brain, along with long-term behavioral alterations that resemble schizophrenia. Such neuroinflammatory states with activated microglia was found to be caused by the elevation of circulating TNF-α, which increased mRNA expression of TNF-α, IL-1β, and KMO in the striatum of amphetamine-sensitized mice, rather than by the direct action of amphetamine. Moreover, behavioral and neuroinflammatory changes in amphetamine-sensitized mice were mitigated by the intravenous administration of hUC-MSC. The immunomodulatory effect of hUC-MSC was found to be associated with the induction of *T*_reg_ and production of the anti-inflammatory cytokine, IL-10. Although in vitro experiments indicated that hUC-MSC conditioned media also inhibited the mRNA expression of TNF-α, IL-1β, and KMO in TNF-α-treated microglia, it is surmised that this effect may last only for short periods, due to the assumption that intravenous hUC-MSCs rarely reach the CNS and do not remain in the body for an extended time. Our findings suggest that even a single intravenous infusion of hUC-MSC may exert a beneficial effect on schizophrenia, by regulating the functional phenotype of microglia and reducing neuroinflammation in the affected brain. Additionally, synaptosome restoration by hUC-MSC in amphetamine-sensitized mice might be associated with the inhibition of synaptic elimination by activated microglia^36^.

Activated inflammatory microglia release pro-inflammatory cytokines such as TNF-α, IL-1β, and IL-6 and the specific influence of these inflammatory cytokines on neurotransmitter systems reportedly leads to schizophrenia and other psychiatric disorders^10,21,37^. The microglia inhibitor, minocycline, produced by the regulation of TNF-α and IL-1β released by activated microglia exerts a certain therapeutic effect on depression and schizophrenia^38^. Activated microglia play a significant role in shifting kynurenine metabolism towards the production of neurotoxic quinolinic acid (QA) by expressing kynurenine 3-monooxygenase (KMO)^39^. Endogenous QA acts as a selective *N*-methyl-d-aspartate receptor (NMDAR) agonist and causes oxidative stress and excitatory neurotoxicity^40^. Besides, dysregulated KMO may contribute to abnormal behavior via QA or kynurenic acid production^39,41,42^. KMO polymorphism (rs1053230) shows a correlation with increased CSF kynurenine acid levels in schizophrenia patients^41,42^. Although the involvement of kynurenine metabolites in the pathogenesis of schizophrenia is not clearly understood, several animal studies have indicated that high concentrations of QA disrupts the neurodevelopmental process and leads to cognitive and behavioral alterations associated with schizophrenia^43,44^. Considering that accumulated metabolites produced by KMO dysregulation are involved in neurotoxicity, increased KMO may be another factor associated with schizophrenia-relevant behaviors in amphetamine-sensitized mice. Because amphetamine itself does not participate in in vitro alteration of TNF-α, IL-1β, and KMO mRNA levels in microglia cells, we hypothesize that sustained elevation of circulating TNF-α may lead to TNF-α-releasing microglia which participate in elevating IL-1β, and KMO levels in the striatum and the hippocampus, resulting in impaired latent inhibition and depressive-like behaviors in amphetamine-sensitized mice, despite amphetamine discontinuation^45–47^.

The activated form of microglia seen in our results may be induced by peripheral TNF-α and lymphocytes^45,47^ which regulate hippocampal slice excitability^46^. The peripheral immune system modulates brain function in various ways and modifies related behavioral patterns^48–50^. Peripheral immune cells or cytokines may enter and exit the meningeal compartment via functional lymphatic vessels connected to deep cervical lymph nodes^51^. Between 20 and 25 kDa of circulating cytokines can enter the brain parenchyma via the para-arterial flux^50,52^ based on reports that approximately 45 kDa fluorescently conjugated ovalbumin tracer can cross paravascular spaces through astrocyte end-feet, into the brain parenchyma within 10 min^53^. Neurovascular endotheliopathy and blood-brain barrier (BBB) hyperpermeability are also observed in schizophrenia patients^45^. T cells and T cell-derived cytokines, such as IFN-γ, in CSF regulate hyperactivity of pyramidal neurons and social deficits^54^ and decreased circulating IL-4 and IL-10 levels are associated with stress vulnerability^34^. Pro-inflammatory cytokines can initiate pathogenic behavior associated with schizophrenia. For example, central injection of tumor necrosis factor (TNF) enhances reactive microglia and produces a prolonged sickness-like behavior^55^. Meta-analysis data indicate that several cytokines including TNF-α levels are elevated in serum of schizophrenia patients^56^. In addition, psychosis in a patient with Crohn’s disease was successfully treated with infliximab, a TNF-α inhibitor^57^. Considered together, sustained elevation of TNF-α in the periphery may induce activated microglia that produce TNF-α.

Most animal studies have first tested intravenously administered MSCs in autoimmune diseases, graft versus host disease and type 1 diabetes mellitus, and in myocardial infarction for immune modulation, and tissue regeneration, respectively, rather than in neurological diseases^58^. This is because MSCs has to be transferred to the target organ in order to be effective, whereas most of MSCs (>80%) administered intravenously are rapidly trapped in the lungs and the time MSCs remain in the lungs reportedly varies from 7 days to 3 months according to the detection method and model used^59^. Our results also indicated that hUC-MSC was not detected in brain parenchyma (data not shown). However, a recent review of mechanisms underlying the effect of systemically administered MSC was focused on its immune-modulatory effect^29,60^. MSC induces T_reg_ through several soluble factors and cell to cell contact^61^. IL-10 is a representative cytokine released by T_reg_^62^. Anti-inflammatory cytokines, IL-10, play a crucial role in suppressing proliferation of activated CD4+/CD25 T cells and neuroinflammation in CNS. A question arises as to whether a single injection of hUC-MSC can achieve sustained elevation of IL-10 and inhibit induction of schizophrenia-relevant behaviors in amphetamine-sensitized mice. Intravenous MSCs can remain in lymph nodes for 96 days^59,63^ and MSC-derived exosomes or mitochondria which carry little nucleic acid and are not detected by qPCR may induce IL-10 releasing regulatory T cells, resulting in a long-term effect on immune-modulatory mechanisms^64,65^. This may explain increased FOXP3 and IL-10 mRNA levels in lymph nodes 30 days following intravenous hUC-MSC injection. Thus, we propose that systemic hUC-MSC administration may play a role as a long-term endogenous T_reg_ and IL-10 inducer.

The anti-inflammatory cytokine, interleukin-10 (IL-10), is associated with schizophrenia and inhibits the inflammatory response, which is in accordance with our in vitro result^66^. A decrease in behavioral abnormalities has been reported in a mouse macrophage-specific IL-10 overexpression model^67^, while a comprehensive meta-analysis has shown that plasma IL-10 levels are significantly reduced in acute recurrent admission schizophrenia patients^68^. Serum IL-10 levels were also associated with the severity of symptoms in schizophrenia patients^10,69^. Treatment with typical neuroleptics, haloperidol, and perazine, normalized the release of IL-1β and TNF-α by inhibiting monocyte activity in schizophrenia patients^70^. Furthermore, other studies have shown that atypical antipsychotic drugs may upregulate IL-10 in schizophrenia^71^. Our in vitro experiment indicated that rTNF-α increased TNF-α, IL-1β, and KMO mRNA expression in microglial cells, which was inhibited by rIL-10. However, MSC-CM mixed with neutralizing IL-10 Ab still inhibited inflammatory function induced by rTNF-α, suggesting that the effect of MSC-CM is associated with other mediators such as TGF-β^30^. In contrast to in vitro study, rIL-10 infusion did not rescue schizophrenia-relevant behaviors in amphetamine-sensitized mice. This might be due to the technical and practical limitation of in vivo IL-10 treatment, which is its short half-life, infusion duration and dose, and complex and intricate immunomodulatory properties^72^. suggesting difficulty in clinical application. This means that hUC-MSC would be superior to rIL-10 in amphetamine-sensitized mice. Nevertheless, we need to consider other possible mechanisms such as various neurotrophic factors released by MSC as well as IL-10 on MSC effect in amphetamine-sensitized mice. Collectively, considering the pharmacokinetic fate of hUC-MSC, the paracrine effect of hUC-MSC acts short-term, and consequently the long-term effect seems to be involved in T_reg_ induction and sustained IL-10 release in amphetamine-sensitized mice.

In conclusion, to our knowledge, the current study is the first study to propose systemic hUC-MSC infusion as a possible therapeutic option in specific schizophrenia group. Although amphetamine models do not represent heterogeneous schizophrenia disorders, based on underling mechanisms of systemic hUC-MSC, we expect stem cell therapy to be effective particularly for patients with elevated TNF-α levels in periphery. The behavioral response to MSC in LI test showed a little bimodal distribution, suggesting setting up precise criteria for MSC treatment in schizophrenia patients would be a critical point when move our findings to clinical trial. Therefore, considering the fact that clinical trials of hUC-MSC have not yet been reported in schizophrenia, our results may be applied to stratified and enriched clinical trial models in schizophrenia. For example, serum TNF-α levels may be a biomarker for application of hUC-MSC in schizophrenia. However, to confirm these findings, further studies that optimize cell numbers, infusion timing, and number of infusions may be needed before moving to the clinical trial phase. Additionally, genetic manipulated hUC-MSC that does not induce circulating IL-10 elevation should be tested in amphetamine-sensitized mice to confirm the mechanism.

## Supplementary information

Supplementary material and methods

Supplementary Figure 1

Supplementary table 1

Supplementary table 2

## Data Availability

The data that support the findings of this study are available from the corresponding author upon reasonable request
