# Supplementary material for: Human umbilical cord-derived mesenchymal stem cells alleviate schizophrenia-relevant behaviors in amphetamine-sensitized mice by inhibiting neuroinflammation
Source: Transl Psychiatry. 2020 Apr 27;10:123. doi: 10.1038/s41398-020-0802-1 (PMC7186225; doi:10.1038/s41398-020-0802-1)
Supplement: Supplementary file 1 — Supplementary material and methods [file 41398_2020_802_MOESM1_ESM.doc]

**Supplementary material**

**Material and Methods**

*1. Behavior assessments*

*1.1. Open field test*

Mice were placed in a 50×50 cm white Plexiglas box, brightly lit by fluorescent room lighting and six 60 W incandescent bulbs placed 4–6 feet above the box. Activity was recorded using a ceiling-mounted video camera and analyzed by Ethovision software (Noldus, Leesburg, VA). The software displays the paths taken by the mice, and measures the total distance moved and the number of entries into the center of the arena (central 17 cm square) in a 10 min session.

*1.2. Light dark exploration*

The apparatus used in this assessment was a box (30×30×30 cm) consisting of one brightly lit open chamber connected to a darkened enclosed chamber. The chambers were connected by a small square hole (7×7 cm). Mice were placed in the corner of the lit chamber, facing away from the dark chamber, and time spent in the dark chamber was manually measured for 10 min.

*1.3. Tail suspension test*

The apparatus consisted of a cupboard with a hook attached to the top. Mice were suspended by securing the tail to the hook by wrapping adhesive tape around the tail. The tail was suspended carefully in a manner that does not to bend the tail, with the tip of the tail wrapped 2 cm away from the top of the hook. Data from mice that climbed up their tails were removed from the test. The time spent immobile during a 7 min testing period was measured by 3 blinded observers.

*1.4. Forced swim test*

Mice were individually placed in a 2 L Pyrex beaker (13 cm diameter, 24 cm height), filled with 23°C water to a depth of 17 cm. All mice were forced to swim for 6 min and the duration of immobility was measured during the final 5 min of the test. Immobility was defined as the time that each mouse spent floating without struggling and only making movements that were necessary to keep its head above water level. The time spent immobile during a 7 min testing period was measured by 3 blinded observers.

*1.5. Three chamber social interaction*

The three-chambered social interaction was adapted from the original method described in a previous study [1]. The testing apparatus consisted of a 60 x 40 cm Plexiglass box divided into 3 chambers as described previously. Mice were able to move between chambers through a small side opening (6 X 6 cm) in the dividers. Plastic cylinders in each of the 2 side chambers contained probe mice or a dummy, and numerous 1 cm holes in the cylinders enabled test and probe mice to contact each other. Mice to be tested were placed in the center chamber and allowed 5 min to explore the box. Unfamiliar, same sex probe mice from the same experimental group were placed in 1 of the 2 restraining cylinders. Time spent in each of the 3 chambers was measured using the EthoVision program, and social preference was defined as (% time spent in the social chamber)/(% time spent in the opposite chamber).

*1.6. Latent inhibition*

This protocol was modeled after a previous study [1]. Each group of mice (control or amphetamine) was randomly subdivided into 2 groups; pre-exposed (PE) and non pre-exposed (NPE). Mice were placed in a shuttle box (Jungdo BIO&PLANT, Seoul, Korea) with a speaker mounted on the back wall. PE mice were exposed to 40 tones (1 tone; 2000 HZ, 30 s duration) separated by 30 ± 40 s, to randomize the intertone interval. NPE mice were placed in the same enclosure for an equivalent amount of time. Immediately following pre-exposure, all mice were given 3 pairing trials of the 30 s tone, followed immediately by a 1 s, 0.3 mA foot-shock delivered through the floor. Pairing trials were separated by 180 s. Mice were returned to the enclosure the next day and presented with an 8 min tone presentation following a 180 s acclimation period. Freezing during tone presentation was measured using the Ethovision program. Data are presented as percentage of the time spent freezing during tone presentation, where LI was defined as the difference in the amount of freezing in response to the tone in PE mice compared with NPE mice. NPE groups were merged for greater statistical power.

*1.7. Acoustic startle/Prepulse inhibition*

PPI testing was modified from a method described by Dulawa [2], and performed in SR-LAB startle chambers (San Diego Instruments, San Diego, CA). Mice were exposed to 5 different types of discrete stimuli or “trials” as follows: a 40-msec broadband 120 dB burst (Pulse Alone trial); 3 different Prepulse + Pulse trials in which either 20-msec long 3 dB, 6 dB or 12 dB above background stimuli preceded the 120 dB pulse by 100 msec (onset to onset); and a No Stimulus trial, during which only background noise was presented. Trials were conducted in a pseudo-random order, separated by an average of 15 s (range: 7–23 s). The test session began with a 5-min acclimation period, followed by 4 consecutive blocks of test trials. Blocks 1 and 4 consisted of 6 consecutive Pulse Alone trials, while blocks 2 and 3 each contained 6 Pulse Alone trials, 5 of each kind of Prepulse + Pulse trials, and 5 No Stimulus trials. Thus, the entire 22-min session consisted of 62 test trials. PPI was calculated as [100 − (prepulse-Pulse trial / Pulse Alone) × 100]. Pulse Alone trials in this calculation were the averaged Pulse Alone values for blocks 2 and 3.

*2. Immunofluorescence*

For perfusion purposes, mice were sacrificed following behavioral assessment. All mice were first deeply anesthetized with pentobarbital (100mg/kg, i.p.) and perfused intracardially with physiological saline followed by ice-cold phosphate-buffered 4% paraformaldehyde (pH 7.4). Each whole brain was dissected and post-fixed in the same fixative for 4 h at 4 °C. Next, brain blocks were cryoprotected in 30% sucrose for 24 h at 4 °C. Twenty-five mm thick sections were obtained using an electronic cryotome. Tissues were washed with PBS and then fixed in 4% formaldehyde and permeabilized with 0.5% Triton X-100 for 5 min. Indirect immunofluorescence was performed using the following antibodies: mouse anti-Iba-1 antibody (1:500; Wako, Cat. # 019-19741) used as the primary antibody. Tissues were incubated in primary antibodies diluted in 0.5% Triton X-100 in PBS containing 10% BSA at 4°C overnight. After rinsing thrice with PBS for 5 min, Alexa 488-(Abcam, ab150105, MA, USA) conjugated secondary antibodies were used for detection. Nuclei were counterstained with 4′6-diamidino-2-phenylindole (DAPI; Sigma, MO, USA). Tissues that were not treated with primary antibodies served as negative controls. Fluorescent images were obtained using a confocal microscope (TCSSP5 II, Leica microsystems, Wetzlar, Germany).

*3. Microglial culture*

*3.1. Rat primary microglial cells*

Primary microglial cells were enriched *in vitro* using the shaking method described in a previous study of ours [3]. Briefly, 2-day-old Sprague Dawley (SD) rats (Orient Bio Inc. Seoul, Korea) were sacrificed and soaked in 75% ethanol for 1 min. Cerebral hemispheres were dissected out following standard techniques and anatomical landmarks, and the meninges were peeled off. The hippocampus, basal ganglion and the olfactory bulb were carefully removed with microsurgical instruments under a microscope, and the remaining cortical tissue was minced with a pair of microsurgical scissors. The shredded tissue was then incubated with 3 mL Hanks' Balanced Salt Solution (HBSS, Invitrogen, USA) for 5 min at 37 °C in a water bath with occasional swirling. After centrifuging at 300 g for 5 min, the cells were plated into 75 cm^2^ flasks coated with poly-L-lysine (Sigma, USA). Mixed glial cells were cultured in DMEM-LG containing 10% FBS and 0.1% Glutamax at 37 °C and 5 % CO_2_ in an incubator. The culture medium was replaced with 15 mL fresh growth medium after 24 h. Subsequently, one-half of the volume of culture medium was replaced with an equal volume of fresh growth medium twice a week. Stratification was reached at the end of this period, and the microglial cells in the upper layer were harvested. On day 11, flasks were placed on a shaking incubator (JEIOTECH, Korea) and shaken for 2 h at 160 rpm and 37 °C. The medium, containing detached microglia, was collected and centrifuged at 190 g for 8 min at 23 °C. Cells were resuspended with microglial complete culture medium (DMEM, 15% FBS, 0.1% glutamax, 5 μg/mL insulin and 1% penicillin/streptomycin) and transferred to PDL-coated plates at a density of 2.5×10^5^cells/ml for qPCR. After serum starvation for 24 h following cell plating for the purpose of attaching cells, the cells were incubated with microglial complete culture medium mixed with 10 μg/mL of amphetamine (Sigma) for 12 h.

*3.2. Murine BV-2 cell culture*

We received Murine BV-2 microglial cells from Dr. Choi HJ in CHA University. BV-2 were maintained in Dulbecco’s modified Eagle medium (DMEM) supplemented with 10% fetal bovine serum and antibiotics at 37 °C in a humidified incubator under 5% CO_2_. Then cells were seeded into 6-well plates at 2×10^5^ cells/well for qPCR analysis. Following serum starvation overnight, the cells were treated with media (control) or 10 μg/mL of amphetamine for 12 h.

*3.3. Mocha cell culture*

Mocha is a Rat Cochlear Microglial Cell Line purchased from Kerafast (Boston, U.S.A). Microglia-enriched cochlear cells were plated in 100 mm cell culture dishes containing DMEM (Gibco) with 10% calf serum (Invitrogen cat. # 16170-078), 1% penicillin/streptomycin (gibco, cat. # 15140122), and antibiotics at 37 °C in a humidified incubator under 5% CO_2_. These cell cultures, which were adherent with a doubling of time for approximately 24 h, were passage 3 times per week with 0.125% trypsin-EDTA. Next, cells were seeded into 6-well plates at 4×10^5^ cells/well for qPCR analysis. Following serum starvation overnight, the cells were treated with media (control) or 10μg/mL of amphetamine for 12 h.

*4. Amphetamine treatment and recombinant IL-10 infusion timeline*

Mice were randomly assigned to control or experimental groups. To induce schizophrenia-relevant behavior, amphetamine (Arlesheim, Switzerland) was injected (3XAMP injection) intraperitoneally three times (09:00, 13:00, 18:00) at a dose of 1mg/kg/day for 6 consecutive days, or administered 3 injections (09:00, 13:00, 18:00) daily, using an escalating dose regimen consisting of 60 injections ranging from 1 to 10 mg/kg. Concurrently, only vehicle was injected into control group mice. Next, recombinant IL-10 (R&D, # 417-ML, 100ng) was infused intravenously once a day (16:00) for 10 days following the final amphetamine injection, and then, a series of behavior assessments were performed.

**Reference**

1. Smith SE, Li J, Garbett K et al. Maternal immune activation alters fetal brain development through interleukin-6. J Neurosci 2007;27:10695-10702.

2. Dulawa SC, Scearce-Levie KA, Hen R et al. Serotonin releasers increase prepulse inhibition in serotonin 1B knockout mice. Psychopharmacology (Berl) 2000;149:306-312.

3. Park MJ, Park HS, You MJ et al. Dexamethasone Induces a Specific Form of Ramified Dysfunctional Microglia. Mol Neurobiol 2019;56:1421-1436.
