# Supplementary material for: Human umbilical cord-derived mesenchymal stem cells alleviate schizophrenia-relevant behaviors in amphetamine-sensitized mice by inhibiting neuroinflammation
Source: Transl Psychiatry. 2020 Apr 27;10:123. doi: 10.1038/s41398-020-0802-1 (PMC7186225; doi:10.1038/s41398-020-0802-1)
Supplement: Supplementary file 2 — Supplementary Figure 1 [file 41398_2020_802_MOESM2_ESM.doc]

**Supplementary Figure 1**

**
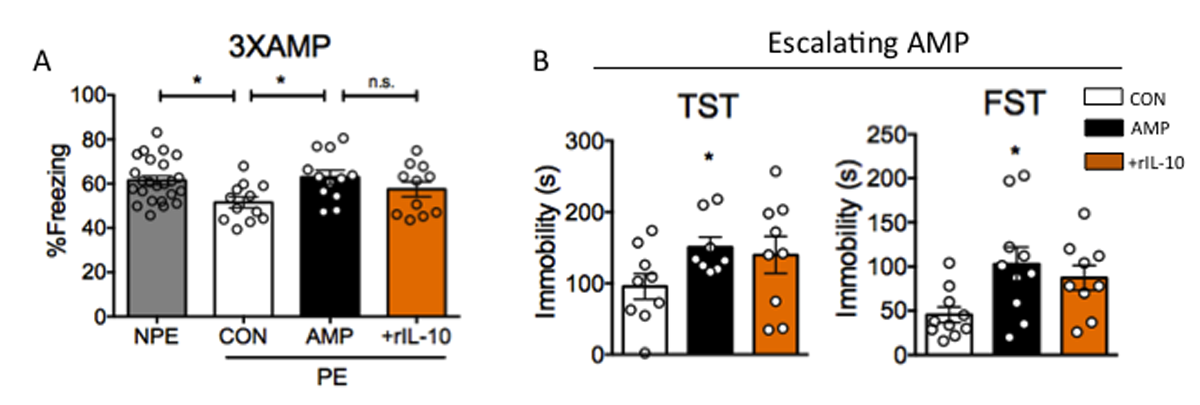
**

**Supplement Fig.1 Effect of rIL-10 in amphetamine-sensitized mice**

1mg/kg of amphetamine was administered intraperitoneally 3 times per day for 6 days (3XAMP) or 3 times injections daily, using an escalating dose regimen consisting of 60 injections ranging from 1 to 10 mg/kg (escalating AMP) was performed for induction of schizophrenia-relevant behaviors. Recombinant IL-10 (100ng, 5ug/kg) was administered intravenously for 10days after amphetamine withdrawal and then LI was conducted in 3XAMP. In Escalating AMP, TST and FST was conducted. (A) n=20 per group, (B) n=10. The data shown as mean ± standard error of the mean (SEM), **p* <0.05, compared with NPE (A) or CON (B)
