# Supplementary material for: Human umbilical cord-derived mesenchymal stem cells alleviate schizophrenia-relevant behaviors in amphetamine-sensitized mice by inhibiting neuroinflammation
Source: Transl Psychiatry. 2020 Apr 27;10:123. doi: 10.1038/s41398-020-0802-1 (PMC7186225; doi:10.1038/s41398-020-0802-1)
Supplement: Supplementary file 3 — Supplementary table 1 [file 41398_2020_802_MOESM3_ESM.docx]

**Supplemenatary Table1.**

**Primer information**

**Mouse, BV-2, SIM-A9**

|  | Forward (5′–3′) | Reverse (5′–3′) |
| --- | --- | --- |
| GAPDH | CATGGCCTTCCGTGTTCCTA | GCGGCACGTCAGATCCA |
| TNF-α | GAGTCCGGGCAGGTCTACTTT | CAGGTCACTGTCCCAGCATCT |
| KMO | CCTGTAGAGGACAATATAGGATCAACAA | GCAAGCCCCATCTACTGCAT |
| IL-1β | GGCTGGACTGTTTCTAATGC | ATGGTTTCTTGTGACCCTGA |
| CX3CR1 | CAGCATCGACCGGTACCTT | GCTGCACTGTCCGGTTGTT |
| CD200R | AAATGCAAATTGCCAAAATTAGA | GTATAGCTAGCATAAGGCTGCATTT |
| IL-10 | CCAGTTTTACCTGGTAGAAGTGATG | TGTCTAGGTCCTGGAGTCCAGCAGACTCAA |
| FOXP3 | GAACCCAATGCCCAACCCTAG | TTCTTGGT TTTGAGGTCAAGGG |
| IL-6 | CCACTTCACAAGTCGGAGGCTTA | GCAAGTGCATCATCGTTGTTCATAC |
| IL-4 | ACAGGAGAAGGGACGCCATG | ACAGGAGAAGGGACGCCATG |
| TGF-β | AGAGGTCACCCGCGTGCTAA | TCCCGAATGTCTGACGTATTG |
| RORγ | CCTGGGCTCCTCGCCTGACC | TCTCTCTGCCCTCAGCCTTGCC |
| IL-17 | CTCCAGAAGGCCCTCAGACTAC | GGGTCTTCATTGCGGTGG |
| P2RY12 | GGGCGTACCCTACAGAAACA | TGTTGACACCAGGCACATCC |
| CD86 | TGTTTCCGTGGAGACGCAAG | CAGCTCACTCAGGCTTATGTTTT |
| ARG | AGGGTCTACGTCTCGCAAGCCA | GGAATCTGCATGGGCAACCTGTGT |
| IFG-1 | AGCAGCCTTCCAACTCAATTAT | GAAGACGACATGATGTGTATCTTTATC |
| TREM2 | TGGGACCTCTCCACCAGTT | GTGGTGTTGAGGGCTTGG |
| iNOS | CATTGGAAGTGAAGCGTTTCG | CAGCTGGGCTGTACAAACCTT |

Rat (Primary cultured microglia, Mocha cell)

|  | Forward (5′–3′) | Reverse (5′–3′) |
| --- | --- | --- |
| GAPDH | TGCTGGTGCTGAGTATGTCG | GCATGTCAGATCCACAACGG |
| TNF-α | CGGTCCCAACAAGGAGGAGAA | AGGAGGGCGTTGGCACGCTGG |
| KMO | TGCTGAGAAATACCCCAATGTG | CTGACAGTTGAATAG. GCTCCATC |
| IL-1β | CATCTTTGAAGAAGAGCCCG | GGGATTTTGTCGTTGCTTGT |
