# Supplementary material for: Human umbilical cord-derived mesenchymal stem cells alleviate schizophrenia-relevant behaviors in amphetamine-sensitized mice by inhibiting neuroinflammation
Source: Transl Psychiatry. 2020 Apr 27;10:123. doi: 10.1038/s41398-020-0802-1 (PMC7186225; doi:10.1038/s41398-020-0802-1)
Supplement: Supplementary file 4 — Supplementary table 2 [file 41398_2020_802_MOESM4_ESM.docx]

|  | **MSC-CM** | **Culture media** | **MSC-CM** | **TNF-α** | **TNF-α+MSC-CM** | **TNF-α+MSC-CM +IL10Ab** | **TNF-α+rIL10** |
| --- | --- | --- | --- | --- | --- | --- | --- |
| **TNF-α**  **pg/mL)** | 5.52 | 11.1±3.22 | 10.15±11.53 | 89076.59±  3758.30 | 29303.22±  1495.42 | 22792.36±  3244.35 | 69303.05±  6777.13 |
| **IL-10**  **(pg/mL)** | 0.61 | 2.24±0.05 | 2.0±0.2 | 1.65±0.79 | 0.61 | 0.2 | 2229.52±  92.71 |

**Microglia**

**TNF-α treated microglia**
